# The first high-density genetic map of common cockle (*Cerastoderma edule*) reveals a major QTL controlling shell color variation

**DOI:** 10.1038/s41598-022-21214-3

**Published:** 2022-10-10

**Authors:** Miguel Hermida, Diego Robledo, Seila Díaz, Damián Costas, Alicia L. Bruzos, Andrés Blanco, Belén G. Pardo, Paulino Martínez

**Affiliations:** 1 Department of Zoology, Genetics and Physical Anthropology, Acuigen Group, Faculty of Veterinary, Universidade de Santiago de Compostela, Campus of Lugo, 27002 Lugo, Spain; 2 The Roslin Institute and Royal (Dick) School of Veterinary Studies, University of Edinburgh, Midlothian, UK; 3 Genomes and Disease Group, Department of Zoology, Genetics and Physical Anthropology, Center for Research in Molecular Medicine and Chronic Diseases (CiMUS), Universidade de Santiago de Compostela, 15782 Santiago de Compostela, Spain; 4 ECOMARE, CESAM-Centre for Environmental and Marine Studies, Department of Biology, University of Aveiro, Santiago University Campus, 3810-193 Aveiro, Portugal; 5 Centro de Investigación Mariña, Universidade de Vigo, ECIMAT, 36331 Vigo, Spain; 6 Mosaicism and Precision Medicine Group, Department of Genetics and Genomic Medicine, The Francis Crick Institute, University College of London, London, UK

**Keywords:** Evolution, Genetics

## Abstract

Shell color shows broad variation within mollusc species and despite information on the genetic pathways involved in shell construction and color has recently increased, more studies are needed to understand its genetic architecture. The common cockle (*Cerastoderma edule*) is a valuable species from ecological and commercial perspectives which shows important variation in shell color across Northeast Atlantic. In this study, we constructed a high-density genetic map, as a tool for screening common cockle genome, which was applied to ascertain the genetic basis of color variation in the species. The consensus genetic map comprised 19 linkage groups (LGs) in accordance with the cockle karyotype (2n = 38) and spanned 1073 cM, including 730 markers per LG and an inter-marker distance of 0.13 cM. Five full-sib families showing segregation for several color-associated traits were used for a genome-wide association study and a major QTL on chromosome 13 associated to different color-traits was detected. Mining on this genomic region revealed several candidate genes related to shell construction and color. A genomic region previously reported associated with divergent selection in cockle distribution overlapped with this QTL suggesting its putative role on adaptation.

## Introduction

The common cockle, *Cerastoderma edule*, is a bivalve mollusc naturally distributed along the Northeast Atlantic coast, from Senegal in the South to Norway and Iceland in the North, inhabiting on intertidal soft sediment regions^1^. This species has an important ecological role on marine sediment renewal and represents a food source for birds, crustaceans and fish, thus playing an important role for coastal ecosystems and marine communities^2^. The species is considered a delicacy and it is commercially fished mainly in Ireland, United Kingdom, Netherlands, France, Spain and Portugal, where it represents a valuable species to coastal fisheries^3^.

As bivalve molluscs, the shell is a fundamental part of the cockle, serving as protection against predators, desiccation at intertidal zones or mechanical damage, enabling behaviours such as being swept by currents or burrowing. This multi-layered exoskeleton is constituted mainly of calcium carbonate deposited into an organic matrix of proteins and pigments secreted by specialized epithelial cells on the dorsal mantle^4^. Although a conserved set of regulatory genes appears to underlie mantle progenitor cell specification, the genes that contribute to the formation of the mature shell are diverse^5^. Technical innovations have allowed to discover surprising patterns of shell pigmentation and rapid divergences in the mix of pigments used to achieve similar color patterns^6,7^. Indeed, the shells of different bivalves are remarkably diverse, and shell pigmentation varies dramatically even within species. Variation in shell color and its pattern may be associated with different biotic or abiotic factors such as predation, substrate, diet or environmental conditions^8,9^. Since color variation has been reported to be controlled to some extent by genetic factors, shell color might be important for adaptation of bivalve population to selective pressures^10,11^. Moreover, as a commercialized food resource, shell color can be important for consumer pleasantness and acceptability, affecting to a certain extent the sale value. Whether common cockle populations show variation in their coloration patterns, if it has an underlying genetic basis and to what extent it could be related to adaptive variation is unknown.

The rapid expansion of next-generation sequencing (NGS) in the last decade has allowed the development of genotyping-by-sequencing (GBS) methods, which have been employed to discover and genotype thousands of single nucleotide polymorphisms (SNPs) in a cost-effective manner, enabling population-scale genetic studies in non-model species^12^. These methods, including Restriction-site Associated DNA (RAD) sequencing^13^ and its derivations, ddRAD^14^, 2b-RAD^15^ or SLAF^16^, have been successfully used for high-throughput genotyping in many aquaculture species^17^, including several important commercial molluscs^18^. GBS methods have been recently applied to understand adaptive variation of common cockle from Northeast Atlantic, and consistent signals of adaptive variation were detected both at microgeographic (dd-RAD;^19^) and macrogeographic (2b-RAD;^20^) scales. GBS have facilitated the construction of high-resolution linkage maps^21–23^, which are important tools for genome scaffolding and assembly^24^ and have aided to disentangle the genetic basis of relevant evolutionary or productive traits through quantitative trait locus (QTL) screening^25,26^. Genetic maps have been used to study the genetic architecture of traits of interest in various bivalve species, such as growth in Zhikong scallop (*Azumapecten farreri*;^27^), bay scallop (*Argopecten irradians*;^28^) or Pacific oyster (*Crassostrea gigas*^29^*;*), various pearl-quality traits in triangle sail mussel (*Hyriopsis cumingii*;^30^) and resistance to pathologies in European flat oyster (*Ostrea edulis*;^31^). Previous studies have also identified QTL for shell coloration in several bivalves including Manila clam (*Ruditapes philippinarum*;^32–34^), hard clam (*Mercenaria mercenaria*;^35^), Pacific oyster (*Crassostrea gigas*;^36–39^), black-lip pearl oyster (*Pinctada margaritifera*;^40^), Akoya pearl oyster (*Pinctada fucata*;^41^) and Yesso scallop (*Mizuhopecten yessoensis*;^42,43^), and therefore, similar strategies might be employed to ascertain the genetic component underlying differences in shell coloration in common cockle.

In this study, we investigated the variation of shell color in Northeast Atlantic populations of European common cockle and studied its genetic architecture through a genome-wide association study (GWAS) on several full-sibs families using the first common cockle high-density linkage map here constructed using 2b-RAD SNP genotyping. A major QTL underlying shell color and its pattern in this species was detected on chromosome 13 and several candidate genes identified. This information should be considered as a potential source for adaptive variation in common cockle and could be exploited in breeding programs to adapt production to consumer demands.

## Results

### Color and pattern variation in common cockle European populations

**Figure 1:**
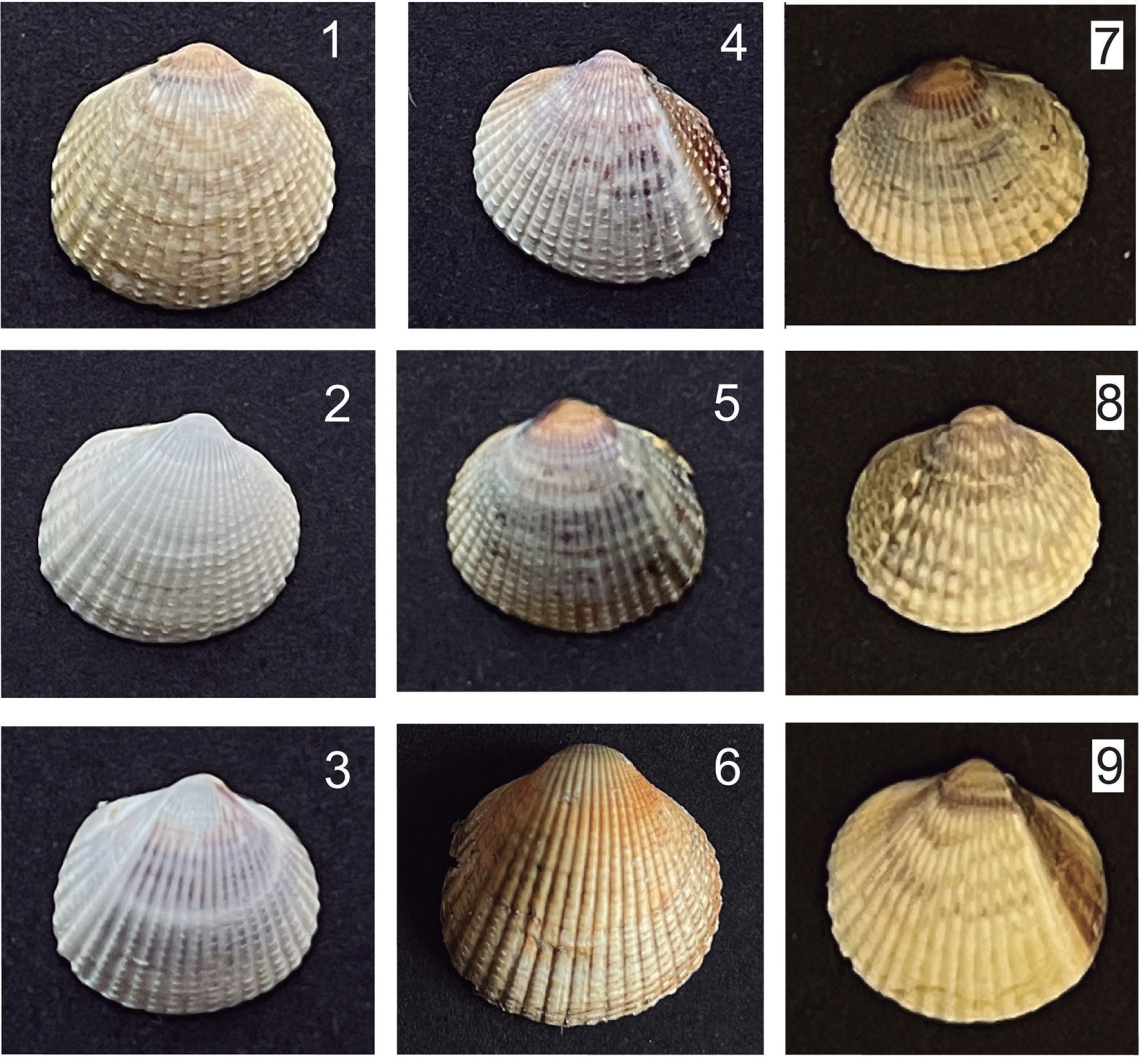
Representative individuals for each color and pattern identified in shells of *C. edule* from Northeast Atlantic. From up to down and left to right: color phenotypes yellow (1), white (2), gray (3), brown (4), black (5), orange (6), and shell patterns circle (7), line (8) and stripe (9).

**Figure 2:**
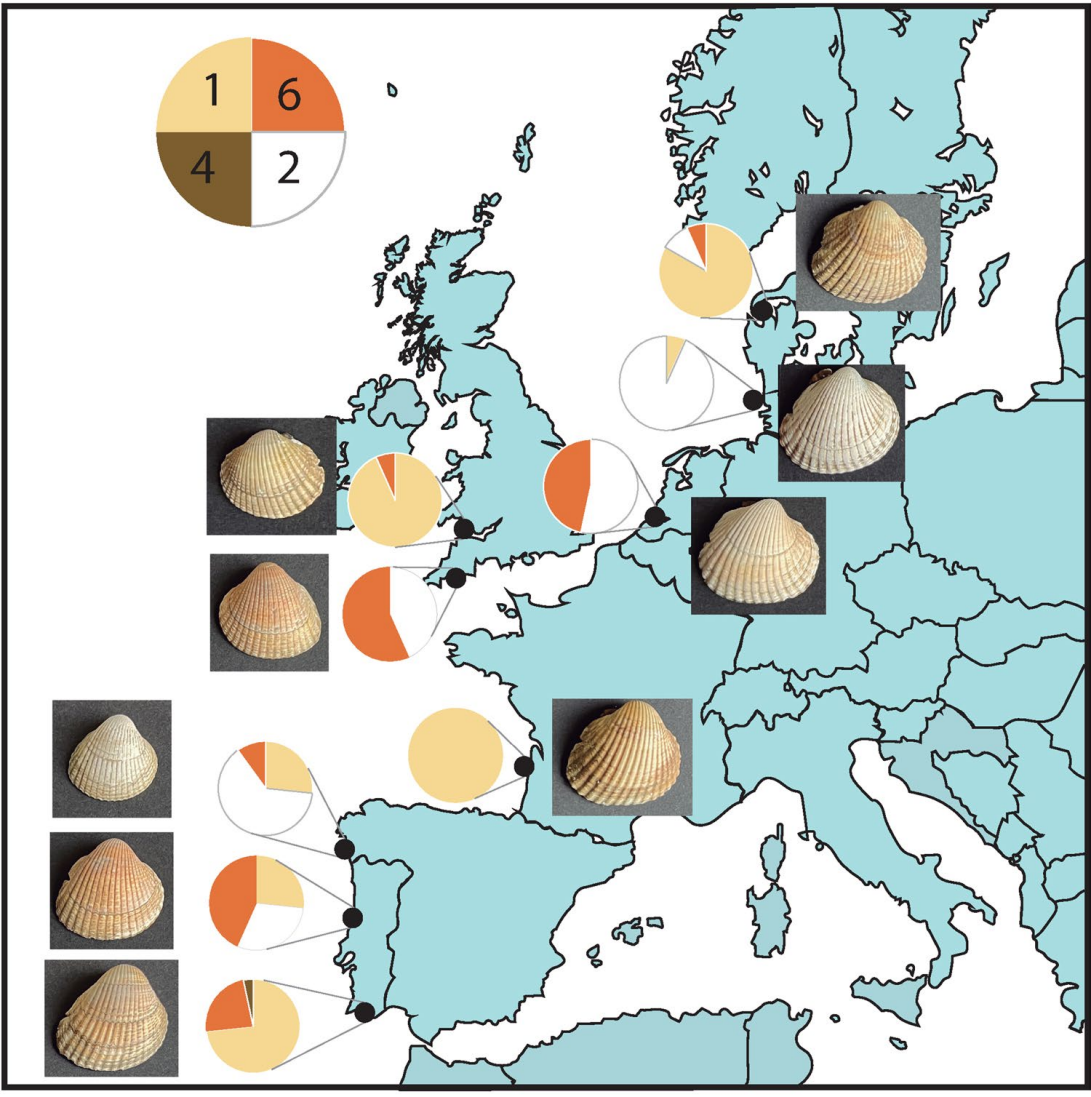
Geographical representation of the European samples of *C. edule*, including pie chart color distributions (yellow (1), white (2), brown (4), orange (6)) and a photograph of a representative individual. From north to south: Nykobing Mors (Denmark), Sylt (Germany), Slikken van Viane (Netherlands), Wales and Plymouth (United Kingdom), Arcachon (France), Baiona (Spain), Aveiro and Algarve (Portugal).

Shell color showed great diversity among the 270 cockle individuals analysed in Northeast Atlantic populations (Fig. 1). Although a predominant color was displayed in each population, important variation was also observed within the nine cockle beds analysed (Fig. 2).

Yellow (1) was the only color detected in the population from France and was the most abundant in the populations from Denmark, Portugal (Algarve) and UK (Wales); white (2) was predominant in populations from Spain, Netherlands and Germany; brown (4) was the least frequent color in this natural survey, only detected in one of the populations from Portugal (Algarve); and orange (6) was rather common in the other population from Portugal (Aveiro), Netherlands and UK (Plymouth) (Fig. 2). Finally, gray (3) and black (5) colors were only detected in the families studied at hatchery using breeders from NW Spain (see below), so apparently rare in European populations.

Shell patterns in wild adults (Fig. 1) were not as marked as in juvenile samples obtained from crosses in the hatchery (see below) and consequently were not systematically recorded. Nonetheless, different color patterns similar to those observed in the hatchery were also identified: (i) a lighter coloration in the circle-shaped umbo in the population from Portugal (Algarve); (ii) a darker band (black or orange) with blurred boundaries in the lateral area of the shell on the opposite side of the ligament in populations from UK (Wales, black) and Portugal (Aveiro, orange); (iii) changes in the arrangement of the periostracum (protein layer) detected in the ventral margin associated with the last growth rings in all populations, excluding Denmark, where remnants of the periostracum were detected on the entire surface of the shell. Additionally, malformation of the shell that affected the last growth rings, likely related to environmental factors, were detected in individuals from France and Portugal (Algarve).

### 2b-RAD sequencing

Cockle breeders to produce families were collected in the natural bed of Noia (NW Spain) and transferred to the ECIMAT-CIM-UVigo (Vigo, NW Spain), where five families were used for GWAS on shell color and its pattern; further, the two more numerous families (F6 and F8) were used for genetic map construction. A total of 275 samples were sequenced in three 2b-RAD libraries: (i) the two parents and 97 offspring of F6; (ii) the two parents and 99 offspring of F8; and (iii) 25 offspring from each of F2, F3 and F7. Around ~ 575 million raw reads were obtained in the first two libraries for the two large families: on average ~ 6.9 million for parents (range: 5,635,542—8,343,112) and ~ 2.8 million for offspring (range: 3,680—5,991,704). After filtering, ~ 75% of the reads were retained and aligned to the cockle genome (Tubío et al., unpublished). An important number of reads were discarded due to mapping to two or more genomic positions (40.74%), resulting in ~ 200 million single-site aligned reads: ~ 3 million from each parent (range: 2,101,828 to 3,843,549) and ~ 900,000 from each offspring (range: 1,317 to 2,158,061).

The third run, with the remaining 75 samples, yielded ~ 275 million raw sequences (~ 3.6 million per offspring), and after the filtering and alignment steps, ~ 92 million high-quality aligned reads were retained (~ 1.2 million per offspring).

### Genotyping and linkage map construction

The *gstacks* module using the *marukilow* model applied to all families yielded 318,755 loci, which resulted in 85,078 polymorphic SNPs using the *populations* module. One individual from each of the two families used for mapping showed a low number of valid genotypes (< 30% of SNPs genotyped) and they were removed. After quality control, 7,094 and 8,439 SNPs were retained for F6 and F8, respectively. The largest number of filtered SNPs in our study was due to missing genotypes and deviations from Mendelian segregation, which can be explained by the presence of null alleles related to polymorphism in the restriction enzyme targets, as previously reported in molluscs^44,45^. There were 1,329 common informative SNPs between the two families, which means that in total 14,204 SNPs were used for the construction of the genetic map.

Separate male and female maps were built for each family (Supplemental Tables S1–S4; Supplemental Fig. 1). To achieve an appropriate number of LGs close to the number of cockle chromosomes (n = 19), different LOD scores were explored for each genetic map (ranging between 7.0 and 9.0), resulting in 21 LGs in all maps. For the maternal map of F6, 3,514 markers were mapped for a total length of 12,572 cM, whereas the paternal map included 3952 markers spanning 16,692 cM (Supplemental Table S5). In F8, 4,698 and 4,368 markers were mapped in the maternal and paternal maps, spanning 25,017 and 23,266 cM, respectively. Shared markers among parental maps were used to build a single consensus map. As a result, 13,874 SNPs were assigned to 19 LGs in the final common cockle genetic map with a total length of 51,778 cM, in accordance with the 19 cockle chromosomes (Supplemental Tables S5–S6). The length of the maps exceeded that expected based on the genome size considering a standard relationship between physical (Mb) and genetic distance (cM) of 1.2 and a C-value of 1.37 pg^46^. The observed elongation of the genetic maps is the consequence of the high number of markers, several mapping families and the limitations of the software used (JoinMap for mapping and MergeMaps for *consensing*), consistent with previous observations^21,47,48^.

**Figure 3:**
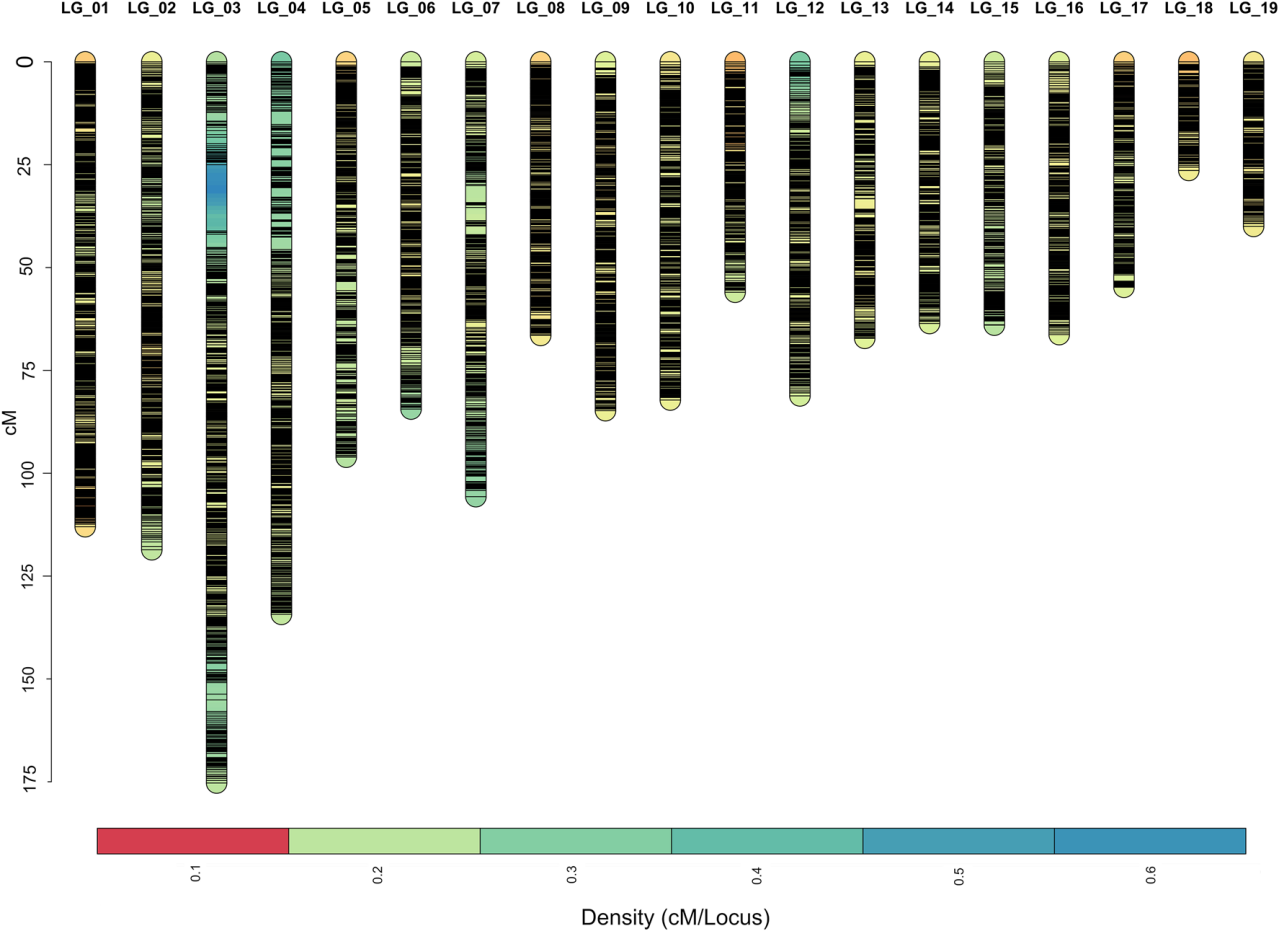
Graphical representation of the consensus map of *C. edule* including 13,874 markers encompassing 1073 cM.. The rule in the left indicates length in centimorgans (cM).

To build a reliable framework genetic map we used the Regression Mapping approach, a similar approximation to that followed in *C. gigas* by Hedgecock et al.^49^. Accordingly, a total of 831 and 340 markers without missing data were selected in offspring of F6 and F8, respectively. Separate maps were built for each parent in each family with a LOD score ≥ 5.0. Graphical representation was not implemented, but the individual groups were merged directly with the “Combine Groups” option of the JoinMap to build a consensus map. The regression of common markers distance was used to correct the distances in the original consensus map to build a new corrected-length consensus map, which included the 19 LGs but with a new total length of 1073 cM (Fig. 3). In the reduced consensus map, the estimated inter-marker distance decreased dramatically (from 4.34 to 0.13 cM), comparable to other genetic linkage maps constructed using 2b-RAD^50,51^, and the average ratio between physical and genetic distance (0.74 Mb/cM) was also much closer to that expected.

### GWAS on shell color type and pattern at hatchery

**Figure 4:**
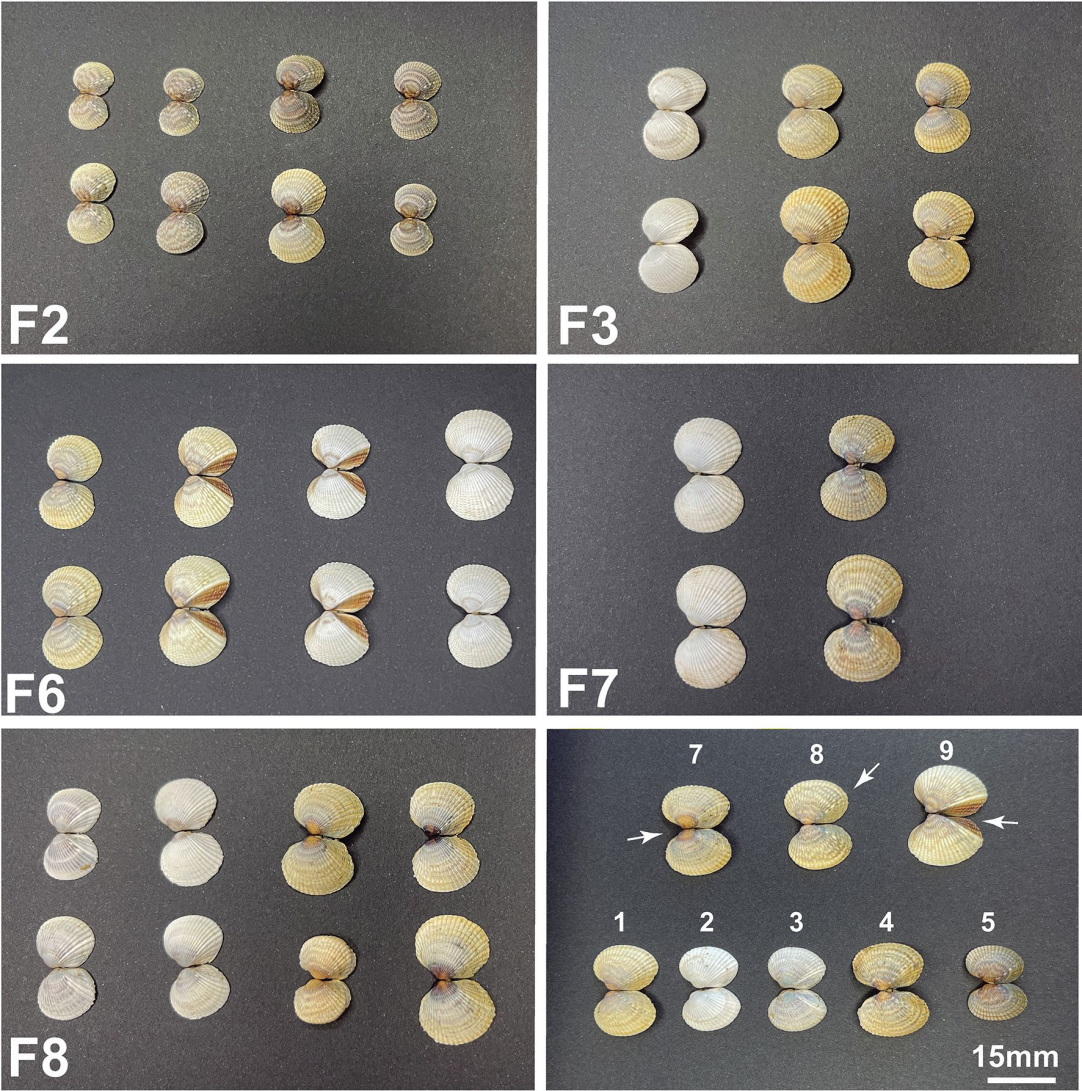
Photograph composition showing representative individuals of the five families (F2, F3, F6, F7 and F8) of *C*. *edule* used for GWAS on shell color and pattern. Last panel: summary of the variation within and between families: yellow (1), white (2), gray (3), brown (4), black (5), circle (7), line (8) and stripe (9).

Shell color and its pattern showed a remarkable variation within and among the five families reared in the same environmental conditions (Fig. 4). Shell color of the offspring of the five families was classified as outlined before (from 1 to 6) and considered as a continuous trait for analyses (Table 1). Five of the six colors identified in common cockle (Fig. 1) were identified in families. Black was the most frequent color (31.6%), but it was detected only in two families (F2 and F8), whereas gray was the least frequent and detected only in F8. White was the only present in all families and the second most abundant in the whole sample (29.8%), and brown was missed only in one family. Orange was not detected in any of the families, only in the wild, as outlined before. Color patterns were only detected in three families and were quite heterogeneous; for instance, stripe was only observed in F6 at a ratio close to 1:1 (Fig. 4; Table 1).

**Table 1:** Distribution of shell color and pattern phenotypes in the offspring of the five families of *C. edule*.

Family	Shell color					Shell color pattern		
	Yellow	White	Gray	Brown	Black	Circle	Line	Stripe
Family 2	3	1	0	8	13	7	11	0
Family 3	7	12	0	6	0	0	0	0
Family 6	24	45	0	31	0	0	0	54
Family 7	0	13	0	12	0	0	0	0
Family 8	4	11	11	0	74	36	8	0
Total (%)	38 (13.9)	82 (29.8)	11 (4.0)	57 (20.7)	87 (31.6)	43 (37.1)	19 (16.4)	54 (46.6)

The estimated heritabilities were high for color and stripe, 0.755 and 0.657, respectively, and moderate-high for circle and line, 0.537 and 0.506, respectively. A genetic correlation of almost 1 was observed between color and circle (0.998), while these patterns showed a moderate negative genetic correlation with stripe (− 0.327 and -0.379, respectively) (Table 2). Line did not show significant genetic correlation with any of the other traits. The circle in the umbo was associated with darker shell colors, which suggests a particular pattern not visible in the whitish phenotypes.

**Table 2:** Phenotypic and genetic correlations (upper triangle and lower triangle, respectively), between color traits of *C. edule* included in this study. (***p < 0.001, *p < 0.05).

Trait	Color	Circle	Line	Stripe
Color		0.465***	0.147*	− 0.501***
Circle	0.998		0.049^n.s^	− 0.208***
Line	0.173	0.137		− 0.130*
Stripe	− 0.379	− 0.327	− 0.217	

**Figure 5:**
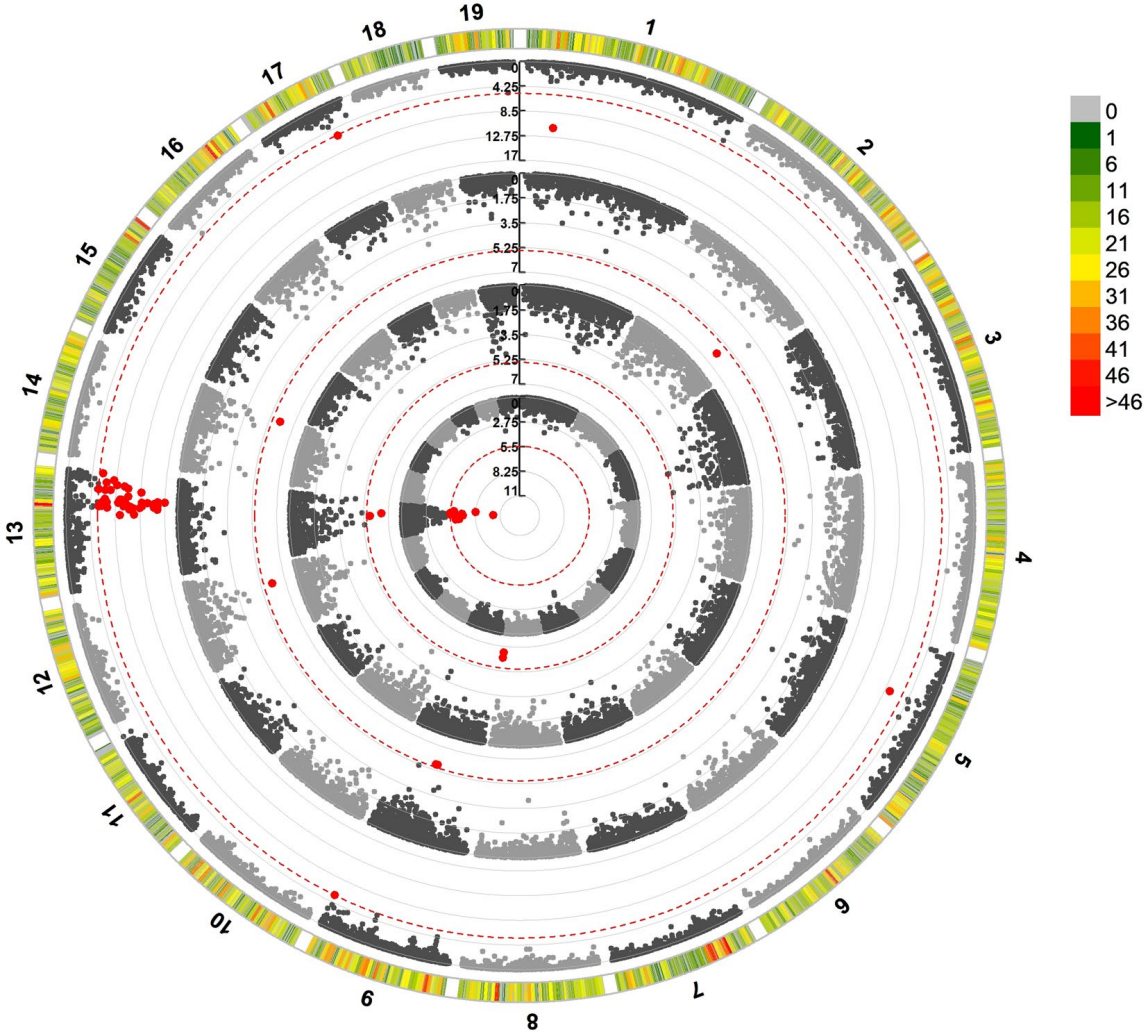
Circular Manhattan plot for SNP significantly associated with shell color and pattern phenotypes in *C*. *edule* based on the Mixed Linear Model (MLM). SNP-GWAS circle representations: inner-most: color; first-middle: circle: second-middle: line; and outer-most: stripe. For Manhattan plot, Y-axis represents − log 10 (*p*-value) of the association with each SNP and X-axis is physical position in bp. The dashed red lines represent the Bonferroni threshold at genome-wide level (*p* ≤ 0.05). SNP markers are represented by gray dots and those above this threshold in red dots.

The complete dataset contained color phenotypes for 275 individuals from five families, genotyped for 13,874 SNPs mapped in the Consensus Map (between 4,643 and 8,439 informative markers per family). GWAS revealed a highly significant genome-wide QTL for color and stripe, and to a less extent for circle, at chromosome C13 (Fig. 5). Other SNP associations at chromosome-level were detected for circle at C8, for line at C2, C9, C12 and C14, and for stripe at C1, C5, C9, and C17 (Supplemental Table S7).

### Gene mining

**Figure 6:**
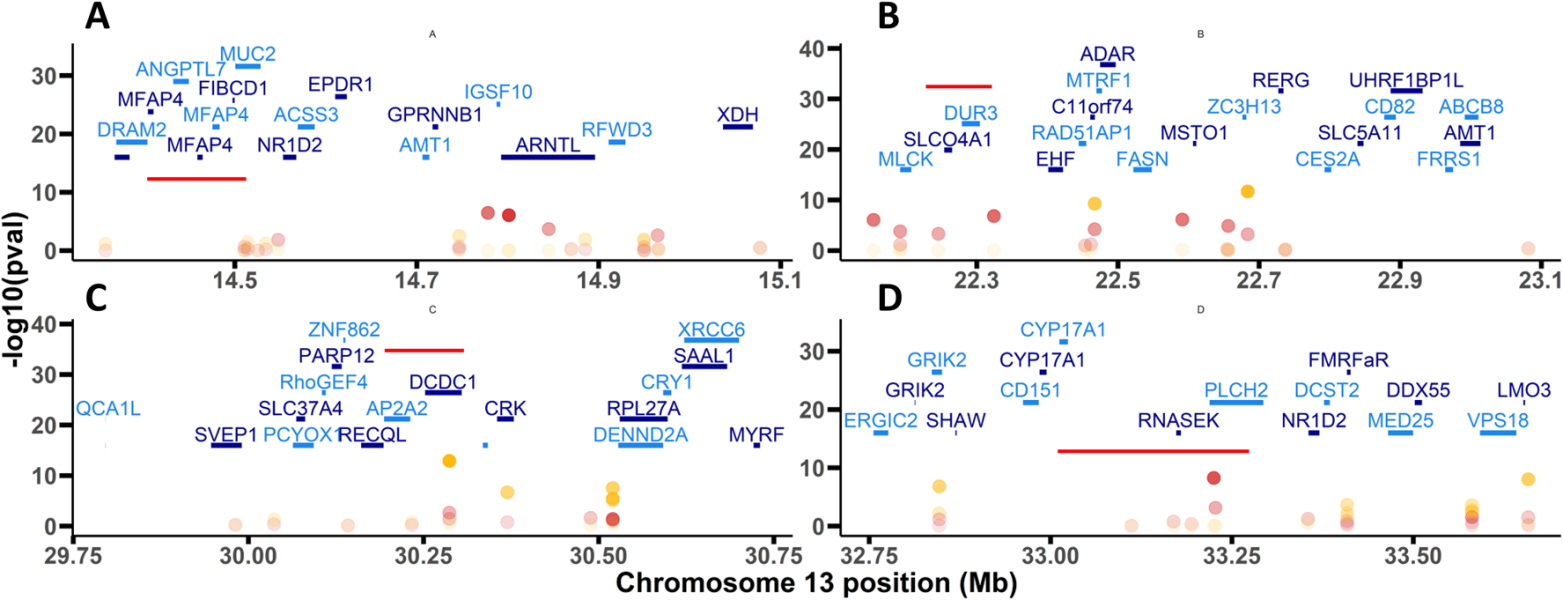
Manhattan plots for SNP significantly associated with color (**A**, **D**) and stripe pattern (**B**, **C**) phenotypes in four chromosome 13 subregions of *C*. *edule* based on the Mixed Linear Model (MLM). Y-axis represents − log 10 (*p*-value) of the association with each SNP and X-axis its physical position in bp. Codes of genes mined in those regions are shown with underlined bars representing their length in Mb; yellow and red dots highlight associated SNPs with stripe and color, respectively; red bars at each plot show the most interesting candidates in the subregion.

GWAS identified a convincing QTL associated with color, stripe and circle located at C13 between 14,367,847 and 33,654,270 bp (Fig. 5). The highest significant SNP associated with the three traits was located at 30,286,849 bp, but across this wide region, there were several stretches defined by highly significant SNPs associated with color and stripe, the most significant-associated traits (Fig. 6). The two end subregions were mainly associated with color, and mining around the most significant SNPs (14,778,145 and 33,225,111 bp ± 500 kb) revealed 16 and 17 annotated genes, respectively (Supplemental Table S7. Half of the genes in the first window, related to shell architecture and color, clustered on a ~ 250 kb region (Fig. 6; Supplemental Table S7): five were related to chitin binding (three microfibril-associated glycoprotein 4, one including a fibrinogen domain, and one DNA damage-regulated autophagy modulator), one to calcium binding (ependymin-related), one to mucin secretion, and one to ammonium transport. The window at the other end (33,225,111 kb) included two genes related to iron binding (two steroid 17-alpha-hydroxylase/17,20 lyase-like) and another one to calcium transport (phosphatidylinositol 4,5-bisphosphate phosphodiesterase).

On the other hand, two consecutive subregions associated with stripe color pattern were located around the most significant SNPs at 22,683,835 and 30,286,849 bp (± 500 kb) on the same chromosome and comprised a total of 36 annotated genes. These group of genes included three cell membrane organic transporters (solute carrier organic anion transporter family member 4A1-like, urea-proton symporter DUR3-like and sodium/glucose cotransporter 4-like); one chitin-binding (sushi, von Willebrand factor type A, EGF and pentraxin domain-containing protein 1-like); several involved in transit across the membrane by endo-exocytosis mechanisms (prenylcysteine oxidase 1-like, glucose-6-phosphate exchanger SLC37A4-like, and AP-2 complex subunit alpha-2-like); and one related to iron chelation (putative ferric-chelate reductase 1) (Supplemental Table S7).

## Discussion

Molluscs represent a highly diverse Phylum of invertebrates comprising an estimated number of 200,000 species, distributed across almost every type of habitat worldwide^18^. They have key roles as ecosystem engineers, water filtering and pollution monitoring, jewelry and, of course, as an important food source. More than 17 million metrics tons of molluscs were farmed worldwide in 2018 and most of this production concentrated in a handful of species of the class Bivalvia^52^. Bivalve aquaculture mainly relies on extensive farming based on the collection of wild seed and harvesting in natural beds, which means that wild populations are under important human alterations^45^.

Shell constitutes a main structure of mollusc anatomy that protects them against predators and desiccation, but that also plays other important functions depending on species. Shells are secreted by the mantle, so color and its pattern are mainly determined by pigments produced by this tissue, although the microstructure of the shell may also contribute to coloration^4,53^. Despite recent efforts, there is still a knowledge gap regarding the genetics underpinning shell color in Mollusca and its potential role on adaptation. While it is well-known that shell color can be under genetic control, biotic and abiotic factors, such as diet, temperature, salinity or pH can also play a role, and the interaction between them is not well understood yet^8^. To address the genetic architecture of complex traits, such as shell color, comprehensive genomics approaches are essential.

Genomic resources have increased exponentially in the last decade as consequence of the lowering cost of sequencing technologies and the new bioinformatic tools that enabled the assembly of genomes at chromosome level, the construction of high-density genetic maps and the genotyping of millions of SNPs for genomic screening^54^. Genetic maps are essential for the identification of genomic regions underlying phenotypic variation for relevant traits under domestic or natural selection^44,45,55^. The first mollusc genetic maps were published in *Crassostrea virginica*^56^ and *Crassostrea gigas*^57,58^ using AFLPs and microsatellites, respectively, but the lowering cost of sequencing technologies enabled genotyping thousands of SNPs for improving map density^29,32^. The common cockle genetic map here constructed comprehends 13,874 markers with an inter-marker distance of 0.13 cM and comprises the 19 expected LGs matching with the haploid karyotype of the species^59^, being, to our knowledge, the densest genetic map published to date in molluscs. Besides its importance for genomic screening, the common cockle genetic map is an invaluable tool for genome scaffolding, as has been previously reported in other species^23,60^, and in fact, five of the seven mollusc genomes assembled at chromosome-level took advantage of high-density linkage maps^45^.

We used the common cockle genetic map to ascertain the genetic component underlying the broad diversity of shell color and pattern on the wild populations of this species in the Northeast Atlantic. Ricardo et al.^61,62^ reported an important variation in shell ion composition in common cockle, apparently related to environmental factors, that allowed tracing back the geographic origin of specimens, but they did not associate this variation with color. Most phenotypes observed in wild populations in our study could be identified in the families produced at hatchery, although their intensity was somewhat faded, likely due to environmental factors operating across the lifespan of the adult specimens collected. Indeed, differentiation of individuals by color in families was very clear, sometimes resembling single gene Mendelian segregation, and heritabilities for all color traits evaluated were high (h^2^ > 0.5), supporting a significant genetic component underlying color variation in common cockle. Both color, circle and stripe showed highly significant genetic and phenotypic correlations, suggesting that the same genes (or genomic regions) could underlie color variation for these three traits. Moreover, the fact that most phenotypes observed in the Atlantic distribution appear to be segregating in a single population from NW Spain supports an important intrapopulation variation in common cockle and suggest that more detailed studies across the full lifespan could disclose more variation than observed in our preliminary screening.

In accordance with these observations, the GWAS performed on five full-sib families produced at hatchery identified a major QTL at C13 for two of the four traits evaluated (color and stripe). This region encompassed ~ 13 Mb, although with different stretches for the same or the different traits studied, which suggests the existence of a broad gene cluster related to color pigmentation in common cockle with different genes playing diverse functions on similar traits. A total of 69 annotated genes were found on this region after mining the cockle genome (Tubío et al., unpublished). We identified a notable proportion of genes related to ion binding and transport/secretion across the cell membrane, such as ammonium or organic transporters, calcium binding or iron chelation, mucin production and several related to endo-exocytosis mechanisms, which play an important role in the development of the shell and that has been related with shell color in other molluscs^35,38,41,42,63,64^. Moreover, shells are secreted by the mantle in a process called biomineralization, where chitin represents an important component^40,65,66^. We could identify six chitin-binding related genes, four of them in a small genomic region encompassing ~ 110 kb including three microfibril-associated glycoprotein 4 genes. Genes related to chitin and calcium metabolism involved in different shell color lines have been reported in *Mizuhopecten yessoensis*^42^. Nevertheless, it is important to note that previous studies have shown that some of the genes related to shell architecture and color are species-specific^8,53^, so further studies should be conducted in the future to ascertain the roles of the genes located at the C13 cluster for a deep understanding of color type and pattern diversity in common cockle.

A reflection on the putative adaptive role of color diversity in the common cockle is worth a final thought. While shell color can be important for the adaptation of bivalve populations to selective pressures, this might not be a major factor in molluscs that live buried in the sediment, such as *C. edule*. Nevertheless, cockles live in the intertidal zone and constitute the feed for different predators, such as birds, mammals and crustaceans, among others. Moreover, a broad color diversity exists in this species across its full distribution range underpinned by a substantial genetic variation, as the high heritabilities estimated in our study demonstrate, so its putative adaptive role should deserve further studies. Interestingly, the C13 color associated QTL overlaps with a genomic region in the same chromosome which showed very consistent signals of divergent selection in the whole Northeast Atlantic and in the Northern Region (above the Ushant Front), including several outliers above the neutral background and highly significant linkage disequilibrium suggestive of selective sweeping^20^.

## Conclusion

Here we presented a high-density genetic map in common cockle, the first reported in the species and, to our knowledge, the highest dense map reported in molluscs to date. The consistency of the map was shown by fitting the number of linkage groups to the haploid chromosome number of the species and by the consistent result of the GWAS on shell color and pattern. This map was used to ascertain the genetic component and architecture underlying the broad color diversity observed in common cockle in the Northeast Atlantic. High heritabilities were estimated for all traits evaluated, supporting an important genetic component underlying coloration pattern. This genetic variation was mainly associated with a single genomic region at C13, where a cluster of genes related to specific enriched functions on shell architecture and color were detected. Our preliminary results in the wild suggest a potential adaptive role of the color variation observed and highlights the importance of deeper studies at population level across the cockle lifespan to understand the significance of the variation observed.

## Material and methods

### Cockle families

In May 2018, 300 mature adult *C*. *edule* cockles were collected in Noia, Galicia (NW Spain) and transferred to the ECIMAT-CIM-UVigo marine facilities (Vigo, NW Spain). Cockles were kept individually in glasses with 0.3L of 1 μm filtered seawater at 20 °C. Spawning was induced by thermal shocks between 10 and 22 °C for 10 h and the quality of the oocytes and sperm was evaluated under a light microscope. Controlled fertilization was carried out by adding sperm to oocytes, one male x one female, at a ratio of 1:10. D-shaped larvae were obtained 24 h after fertilization with a transformation rate from trochophore larvae of 42% ± 19. Following this protocol, a total of eleven full-sib families were obtained by crossing one male x one female, involving a total of 11 females and 7 males.

Larvae from each family were cultured in individual 150 L cylindrical-conical tanks at a density of 8 ± 3 larvae mL^−1^ with sea water filtered at 1 µm and treated with UV, slight aeration, and temperature 19.0 ± 1.4 °C in an open circuit with a renewal of 5% volume / hour. The diet consisted of *Tisochrysis lutea* (ECC038), *Chaetoceros neogracile* (ECC007), *Phaeodactylum tricornutum* (ECC028) and *Rhodomonas lens* (ECC030) in a ratio of 1:1:1:1 (according to the cell count), and *Tetraselmis suecica* (ECC036) was included from the seventh day of culture. The daily diet was administered automatically every 4 h in 6 daily intakes, maintaining a constant concentration in the tank of 20–40 cells µl^−1^. At 14 days post-fertilization (dpf), pediveliger larvae from each family were transferred to separate 50 L tanks in suspended baskets with constant aeration, temperature 18.4 °C ± 0.5 and a renewal rate of 50 L day-1. The animals were fed with the same diet as described above but maintaining a constant density of 168 ± 48 seeds cm^2^. Metamorphosis of larvae took place in those tanks.

At 112 dpf one hundred individuals from each of two families, F6 (12.74 ± 0.73 mm) and F8 (12.23 ± 1.48 mm), were selected for genetic mapping and for GWAS on color patterns, while twenty-five individuals from each of three additional families, F2 (9.82 ± 0.85 mm), F3 (12.58 ± 0.90 mm) and F7 (13.12 ± 1.67 mm), were sampled for increasing statistical power of GWAS. The meat of each individual was fixed in pure ethanol and sent to the Genomics Platform of University of Santiago de Compostela (Campus de Lugo) for DNA extraction.

### Color variation of common cockles

After the visual inspection of all the shells in the study, shells were classified according with their color into six phenotypic classes: yellow (1), white (2), grey (3), brown (4), black (5), orange (6). Further, specific color patterns of the shell were consistently identified and recorded as three other traits: (i) a circle in the umbo, differentiated from the rest of the shell and generally yellow (circle); (ii) a broken white line (line); and (iii) a stripe with white line edge (stripe) (Fig. 1). A typical individual from each class was selected to define a standard pattern to categorize the phenotype of each shell in the study. This evaluation was performed by a single observer, who revised and scored all the shells in two independent rounds. Accordingly, color and pattern of the shell were assigned to all hatchery specimens (F2, F3, F6, F7, F8 families), while mainly only the color was assessed in the European wild population samples provided by the Scuba Cancers Project (ERC-2016-STG). Despite similar color patterns were detected in the hatchery and wild individuals, its classification was not straightforward in wild specimens, likely due to environmental factors across their life span, and therefore they were not systematically recorded.

### 2b-RAD library construction and sequencing

DNA was extracted from the whole meat using the E.Z.N.A. E-96 mollusc DNA kit (Omega Bio-tek) following manufacturer recommendations. Library preparation followed the 2b-RAD protocol^15^ with slight modifications^21^. Briefly, DNA samples were adjusted to 80 ng µL^−1^ and digested using the IIb type restriction enzyme *AlfI* (Thermo Fisher). As a result, the genome was cut in fragments of 36 bp of length, with the restriction enzyme recognition site in the middle. Specific adaptors, also including individual sample barcodes, were ligated and the resulting fragment amplified. After PCR purification, samples were quantified using Qubit 2.0 fluorometer (Life Technologies, Carlsbad, CA, USA) and equimolarly pooled. The pools were sequenced on a NextSeq 500 Illumina sequencer using the 50 bp single-end chemistry in the facilities of FISABIO Sequencing and Bioinformatics Service (Valencia, Spain). The two bigger families including ~ 100 offspring per family, with parents at double concentration, were multiplexed each in one run, whereas the other seventy-five samples, without parents, were multiplexed in a third run.

### Data filtering and genotyping

Raw reads were first demultiplexed according to the individual barcodes ligated during library construction. Then, reads were filtered in three consecutive steps: (i) trimmed to 36 nucleotides (length of *AlfI* generated fragments) and discarding reads below this length; (ii) removing reads without the *AlfI* recognition site in the correct position; and (iii) removing reads with uncalled nucleotides or a mean quality score below 20 in a sliding window of 9 nucleotides. Custom Perl scripts (available on demand) were used in the first two steps, and the *process_radtags* module in STACKS v2.0^67^ was used for the latter (-c -q -w 0.25 -s 20). Bowtie 1.1.2^68^ was used to align the filtered reads against the recently assembled reference genome of the species (Tubío et al., unpublished), allowing three mismatches and a unique alignment (-v 3 -m 1 –sam), so reads aligning to two or more sites were discarded. The *sam* output files were converted to *bam* files and appropriately sorted to feed the *gstacks* module in STACKS, using the *marukilow* model to call variants and genotypes. The collection of putative SNPs was exported using the *populations* module.

### Linkage map construction

Files containing the putative SNPs of the two families selected for genetic mapping (F6 and F8) were processed to conform an appropriate dataset for mapping. Those SNPs not informative in the parents of each family or with missing data in one or both parents were filtered, as well as those SNPs with extreme deviations from Mendelian segregation (*p* < 0.001) or genotyped in less than 60% of the offspring.

Genotypes of retained SNPs were properly coded as Cross Pollinator (CP) cross type with unknown linkage phase to build genetic maps using JoinMap 4.1 genetic mapping software^69^. Firstly, markers were associated to their linkage groups using the Grouping function of JoinMap based on a series of LOD scores increasing by 1, from 4.0 to 9.0. The LOD score was then selected in each family based on the number of chromosomes of the common cockle karyotype^59^. Markers in linkage groups with less than 10 markers and unlinked markers were excluded for further analysis. Secondly, marker ordering was performed using the Maximum Likelihood (ML) algorithm implemented in JoinMap. Default parameters were used except the chain length that was increased from 10,000 to 20,000, and multipoint estimation of recombination frequency were in general increased to ensure convergence: length of burn-in chain, 20,000; number of Monte Carlo EM cycles, 10; and chain length per EM cycles, 5000. The *Kosambi* mapping function was used to compute centiMorgans (cM) map distances in individual female and male maps in each family. Finally, female, and male consensus maps (from both families), and a species consensus map were built using MergeMap^70^.

The combination of the ML mapping algorithm implemented in JoinMap with missing data and genotyping errors, as well as the use of MergeMap to merge the parental maps and the large number of markers, can derive in inflated map lengths (see e.g.^71–73^). Nevertheless, ML is more powerful and robust in ordering markers in CP populations compared to Regression Mapping (RM)^74^. To obtain the most accurate linkage maps, a mixed strategy was implemented: (i) a framework genetic map was constructed using RM only with markers without missing data; (ii) then, a linear regression between ML and RM distances using the same marker pairs was performed; and (iii) this value was used to adjust the map distances in the ML ordered consensus maps. All maps were drawn using MapChart ver. 2.3^75^ and LinkageMapView^76^.

### GWAS analysis

For the study of the genetic parameters underlying color variation, the information of five families was used to estimate genetic correlations and heritabilities using hiblup v1.3.1 (https://github.com/xiaolei-lab/hiblup). The same set of families was used for a genome wide association study to ascertain the association between phenotypic traits and SNPs across the cockle's genome. The genotypes of mapped markers in the Consensus Map and the phenotypes of offspring were used to look for association in the two families used for mapping (F6 and F8), but in addition, to increase statistical power, the same markers were used in three additional families (F2, F3 and F7). In this case the genotypes were also filtered by minimum allele frequency (–maf 0.05) and minimum number of genotypes (–geno 0.5) using PLINK 1.9 (www.cog-genomics.org/plink/1.9;^77^).

A Mixed Linear Model^78^ was applied to the complete dataset of filtered genotypes and the corresponding color phenotypes catalogued as mentioned above using rMVP^79^, a parallel accelerated tool for GWAS implemented in R^80^. The three color pattern traits (circle, line and stripe) were coded as binomial traits (presence /absence). The kinship matrix was previously computed following VanRaden^81^, and the EMMA method was employed to the variance components analysis^82^. rMVP and the *qqman* R package^83^ were used to plot the results.

### Gene mining

Coding genes included in a genomic window defined around the most significant SNPs (± 500 kb) detected in GWAS for each trait were retrieved using the common cockle genome (Tubío et al., unpublished).

## Supplementary Information

Supplementary Figure 1.

Supplementary Information 2.

Supplementary Information 3.

Supplementary Information 4.

Supplementary Information 5.

## Data Availability

2b-RAD sequencing data of cockle’s families are linked to the SRA project PRJNA862862 and will be released on 2022–11-01, available at the following link https://www.ncbi.nlm.nih.gov/sra/PRJNA862862 (temporary submission ID: SUB11861213).
