# Supplementary figures and images for: The first high-density genetic map of common cockle (Cerastoderma edule) reveals a major QTL controlling shell color variation
Source: Sci Rep. 2022 Oct 10;12:16971. doi: 10.1038/s41598-022-21214-3 (PMC9551087; doi:10.1038/s41598-022-21214-3)

## Slide 1
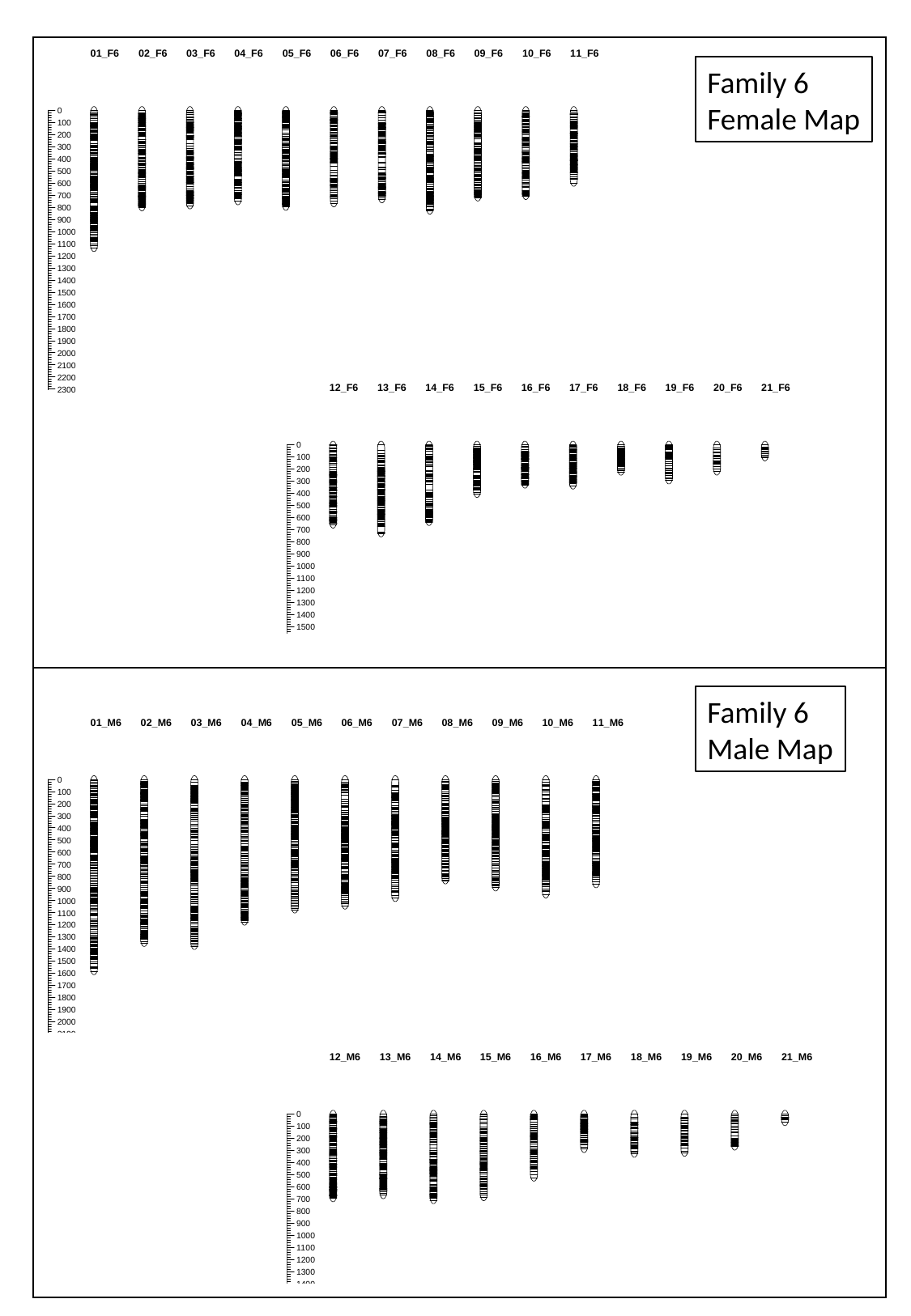

Family 6
Female Map
Family 6
Male Map

## Slide 2
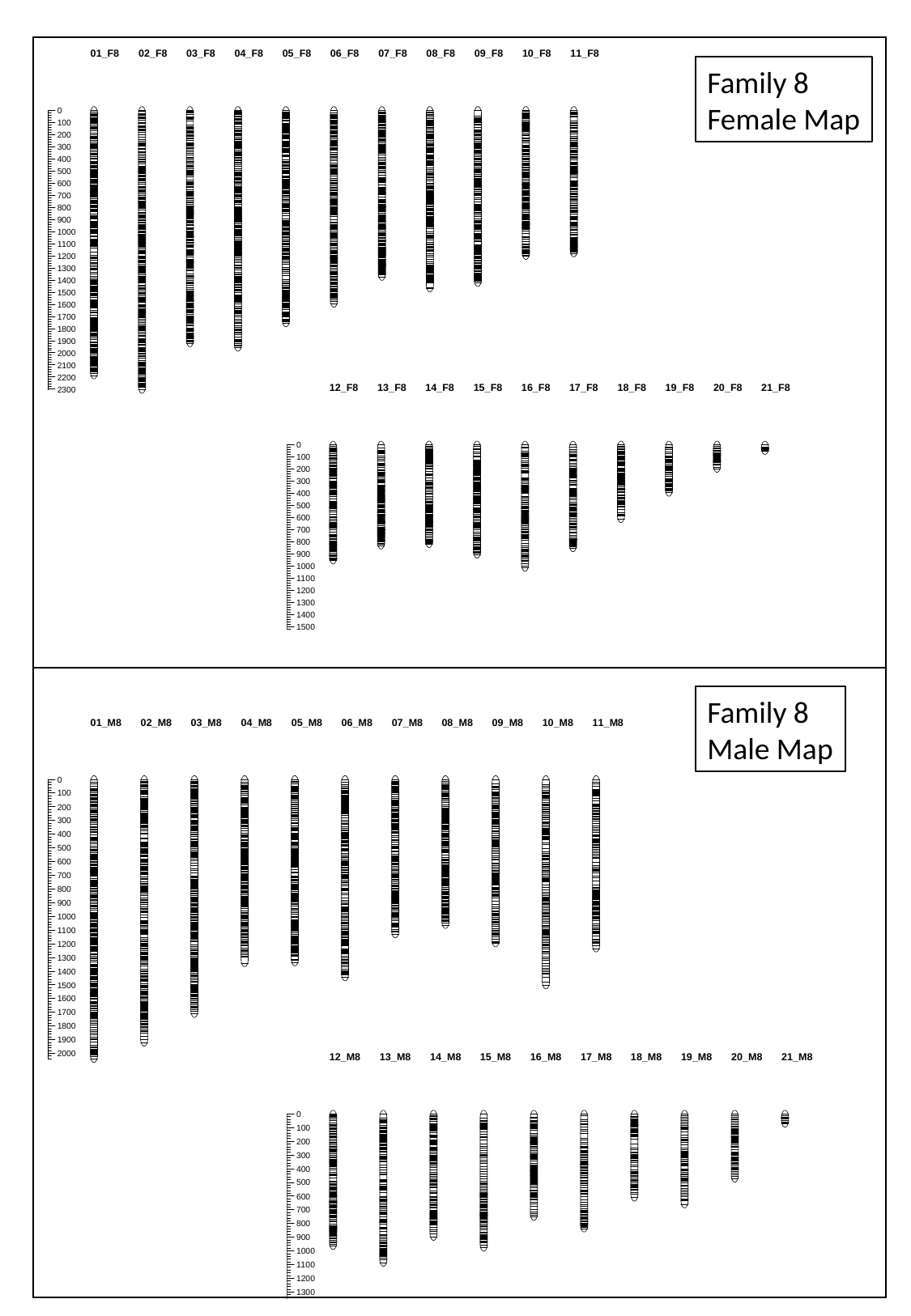

Family 8
Female Map
Family 8
Male Map

Supplement: Supplementary file 1 — Supplementary Figure 1. [file 41598_2022_21214_MOESM1_ESM.pptx]
